# Supplementary material for: The Generalized Matrix Decomposition Biplot and Its Application to Microbiome Data
Source: mSystems. 2019 Dec 17;4(6):e00504-19. doi: 10.1128/mSystems.00504-19 (PMC6918030; doi:10.1128/mSystems.00504-19)
Supplement: TABLE S1 [file mSystems.00504-19-st001.pdf]

mSystems Submission Template mSystems Submission Template mSystems Submission Template mSystems Submission Template mSystems Submission Template mSystems Submission Template

GMD		AMD		SVD	
taxa	rank	taxa	rank	taxa	rank
halophilus	26	succinus	137	succinus	137
alloiococcus	22	lactobacillus	67	aerococcaceae3	134
aerococcaceae1	64	aerococcaceae3	134	corynebacterium	50
tetragenococcus	33	corynebacterium	50	staphylococcus	139
aerococcaceae3	134	stationis	15	alloiococcus	22
stationis	15	enterobacteriaceae	12	enterobacteriaceae	12
enterobacteriaceae	12	halophilus	26	staphylococcus	161
aerococcaceae2	42	alloiococcus	22	halophilus	26
yaniella	24	acetobacter	131	aerococcaceae1	64
granulicatella	48	yaniella	24	enterobacteriaceae	17
avg. rank	42	avg. rank	61.8	avg. rank	76.2
