# Supplementary figures and images for: The Generalized Matrix Decomposition Biplot and Its Application to Microbiome Data
Source: mSystems. 2019 Dec 17;4(6):e00504-19. doi: 10.1128/mSystems.00504-19 (PMC6918030; doi:10.1128/mSystems.00504-19)

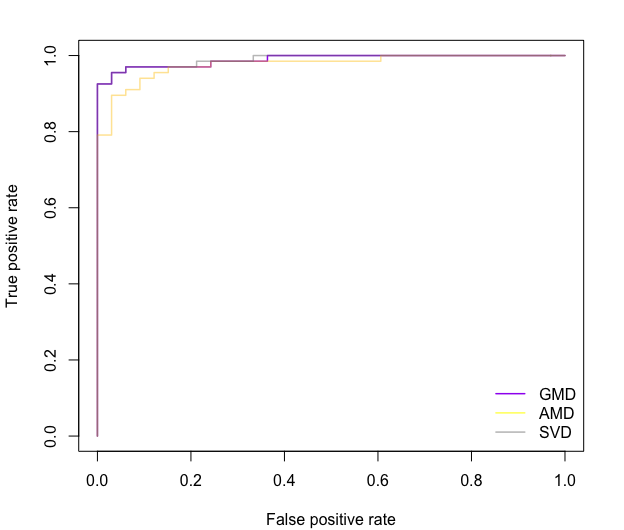

Supplement: FIG S1 [file mSystems.00504-19-sf001.tif]
